# First impressions of the Fore

**DOI:** 10.1098/rstb.2008.4017

**Published:** 2008-11-27

**Authors:** Shirley Lindenbaum

**Affiliations:** Graduate School and University Center, City University of New York365 Fifth Avenue, New York, NY 10016-4309, USA

On this day of celebration and historical memories I thought it would be interesting to reflect on what I had written in my field diary during the first few weeks of anthropological research, which Robert Glasse and I began in July 1961. What were our first impressions of the Fore, and what were the first interactions between the Fore and ourselves?

After we stopped overnight at Okapa in the North Fore, Mert Brightwell, the district officer, lent us his driver and Landrover to explore the South Fore, where we hoped to choose a suitable field site. Epidemiological reports had indicated that this was where the incidence of kuru was at that time the highest. Leaving Okapa we passed over the ridge dividing North and South and continued for several miles until we found the road blocked by a party of men. Alerted by the rare sound of an approaching vehicle, which could be heard for approximately 20 min before it reached them, they wanted to know who might be entering their region. After several minutes of animated conversation, during which we conveyed why we had come, they announced that we should go no further. We should stay with them at Wanitabe in the central South Fore.

This first impression of the Fore eager to engage with new people, new things and new sources of information was one that would last. The Fore never wavered in their welcome, weaving us into their community through the exchange of food, goods and labour, and the sharing of information. As we visited the hamlets small groups of inquisitive young men tagged along. Women, young and old, were always busy in the gardens. We benefited, perhaps, by being in the ambiguous category of outsiders—not government officers or missionaries, who they had encountered already, but people who wanted to learn about their lives. At first, they called us ‘story masta’ and ‘story missus’, and later by our names, which were soon given to several infants (eliciting birth gifts from us).

After the road stop, we accompanied them to their village settlement where we were shown a small bamboo and thatch structure they had just built for Sunday church services. This is where we lived for several weeks until, with their help, we constructed a more permanent house with a large outer room open to all. Here, people came to talk, shelter from the rain, and roast sweet potatoes on the pot-bellied stove. So, what did I write in my diary?

My first entries record the supplies provided for us by the Department of Health, a list that will evoke nostalgia for old field hands in today's audience:

four large and one small patrol boxes; two boilers; one large and one small camp oven; one hurricane lamp; three patrol tables; two canvas chairs; one shower bucket; one tin dish; two bedsails; one saucepan.

The Fore had lined up at the open door of the little ‘church’ to inspect all this cargo.

We also took with us a typewriter, a camera and a klunky tape recorder, which would seem archaic to contemporary fieldworkers who now have digital cameras, solar-operated computers and mobile phones.

The next diary entries consist of word lists—pages and pages of Fore words for edible and inedible food, the names of plants and trees, body parts, dress and ornaments, place names, people they considered to be kin, and what kind of relationships these were. I recall one tender moment when a man who was to become a close friend was providing us with information about marriage arrangements. (The Fore have a preference for cousin marriage—for men to marry their Mother's Brother's Daughter.) Interrupting our questions, my friend asked me if I had married my cousin. ‘No’, I said, ‘I married an unrelated person’. Placing his hand on my arm he said, ‘Oh, I am so sorry’. I understood then something about the emotional valence of relatedness in small kinship-based communities. Two days after the last of the word lists I made a note that a certain man brought us a bunch of bananas but would not accept payment. I took this to be our first entry into an important social relationship in this pre-capitalist, exchange-based economy. The bananas would later require a reciprocal gift.

A curious entry the following week just says, in capital letters, JOKE. I then record a humorous story told by one of our translators about a small boy who had gobbled down his food, but choked on a piece of pork. The story was met with roars of laughter by others listening to our conversation. I thought then that I had recorded the Fore version of ‘the man who slips on a banana skin’—although the Fore story may have also been about inappropriate greed. In retrospect, it now seems surprising that despite the aura of death resulting from their daily encounters with kuru, the Fore were often playful, not dour and forbidding as some other highland people have been described.

The most detailed entry during these early days concerned a death payment held at the neighbouring settlement of Amora. An epidemic of illness rippling through the pig population, probably anthrax, had provoked rapid payment of kinship debts before the pig herds were depleted. Memorial feasts were held before the death of old people, who sat watching their own mortuary ceremonies, listening to the oratory. The events did not seem gruesome. People said, in fact, that the old people's ‘bellies would be warmed’ by seeing how much cargo was distributed in their names. Again, it seemed I was recording something about the culture of the emotions.

Some days later, a party of Awa people arrived hoping to sell their black palm bows. (The Awa, who speak a different language, live 2 days' walk to the south.) I see that I bought two bows and gave them to two luluais (government-appointed leaders), keeping up exchange relations. A few days later, another Awa party arrived with more palm bows, and again I offered ‘shillings’, the adopted Australian currency at that time. The man I approached responded with anger, threw the money to the ground and spat on it. He wanted salt. This seemed to provide a small glimpse into pre-colonial Awa–Fore trade relations. (The Fore had manufactured and traded potassium-based salt for forest products.)

Later, in early August, I have detailed notes on a marriage payment (bride price) at which the bride and groom were not present. The bride price consisted of two piles of food, as well as locally made bark cloaks, vegetables, some shells and 15 pounds (approx. $20 US). By the late 1990s bride price had reached stunning levels, some payments rising as high as 5000 Papua New Guinea kina (in 1990 1 kina =$1.10 US), piles of vegetable food, but no bark cloaks. Boxes of imported frozen lamb parts now replaced pigs. By mid-August 1961 it was clear who the local ‘bigmen’ (leaders) were. Significantly, this short list included the man who had given us the bunch of bananas during our first few days.

Our plan had been to begin investigating kuru not with questionnaires, but by observing how those with kuru were treated, and listening to the way people talked about the disease. By early August, however, we were aware that we were not seeing women with kuru. In a quest for a cure, the sick women, accompanied by male kin, had left Wanitabe, crossing the Yani River and mountain range to the west, to reach Gimi territory. We decided to follow. After 2 days' walk, we reached the Gimi settlement at Uvai where the healer had established his base. My diary indicates that Gimi women rubbed my breasts and Robert's shins. Men did not shake hands, which appeared to be a colonial acquisition.

In separate typed pages I describe the long structures housing the Fore patients, imitations of the Okapa ‘house sick’ (hospital). The women hoping for a cure were a poignant sight, their shoulders scarred with the welts of the healer's blood-letting procedure using a small glass-tipped arrow. On our sad journey back to Wanitabe by a slow southerly route, we stopped from place to place and heard stories of treatment failure. Later, some people called the healer a ‘fraud’, interested only in money. However, the Fore never lost hope that a cure could be found. When the same man came through the South Fore some months later, people sought him out again. His cures, they said, were effective. Sorcerers had ‘poisoned’ the women a second time. Although we did not share their theory of disease causation, the search by the Fore for an effective therapy seemed much like the hopeful journeys made by patients in our own society.

This record of first impressions may seem to be a list of random observations. Reading them again last week, however, I recaptured the image of the people who told us these things. Moreover, the information came back with a particular intensity, accompanied by the sounds and smells of the days when they were recorded. I was struck also by the importance of ‘first impressions’. In a short time we had learned something about kinship and marriage, sickness and death, political leadership, the importance of exchange relationships, trade partners, gender relations, beliefs about the cause of disease, and the way in which different cultures evoke the emotions. I was reminded of what people said about D.H. Lawrence's autobiographical ‘novel’, *Kangaroo*, which he wrote in 45 days shortly after arriving in Sydney. First impressions seem to be keen and intense, something lost when the strange becomes familiar.

One last thought. After this London meeting it would be wonderful if some of the Fore felt inspired to write down their own memories of these amazing years.

